# Full-term pregnancy despite severe hypophosphatemia caused by tumor-induced osteomalacia

**DOI:** 10.1093/omcr/omae125

**Published:** 2024-10-26

**Authors:** Thien Binh Nu Truong, Anh Trong Nguyen, Luong Dai Ly

**Affiliations:** Department of Pediatrics, School of Medicine, Vietnam National University Ho Chi Minh city, YA1 Administrative Building, Hai Thuong Lan Ong Street, Dong Hoa Ward, Di An City, Binh Duong Province 75308, Vietnam; College of Health Sciences, VinUniversity, Vinhomes Ocean Park, Gia Lam District, Hanoi 12400, Vietnam; Department of Physiology, School of Medicine, Vietnam National University Ho Chi Minh city, YA1 Administrative Building, Hai Thuong Lan Ong Street, Dong Hoa Ward, Di An City, Binh Duong Province 75308, Vietnam

**Keywords:** tumor-induced osteomalacia (TIO), hypophosphatemia, fibroblast growth factor-23 (FGF23), pregnancy, child growth

## Abstract

A woman in her 30s with a history of multiple bone fractures unexpectedly became pregnant and delivered a full-term baby through cesarean section, despite suffering from excruciating pain without any apparent cause or specific treatment. The patient was referred to our endocrine clinic following childbirth. Blood tests revealed a life-threatening low level of serum phosphate, normal 25-hydroxy vitamin D concentration, low TmP/GFR ratio, and elevated FGF23 levels. A PET/CT scan with Gallium-68 Dotatate identified an abnormal tumor in the right calcaneus. Her FGF23 level returned to normal soon after surgical removal of the tumor, which was confirmed to be chondroblastoma. Her child’s cognitive and motor skills typically developed from the newborn to preschool age. He exhibited excellent long-term growth, without any signs of rickets.

## Introduction

Tumor-induced osteomalacia (TIO) is a debilitating syndrome caused by excessive secretion of fibroblast growth factor 23 (FGF23) from phosphaturic mesenchymal tumors, and rarely as a non-metastatic manifestation of other neoplasms. The lower limbs are the most common location of tumors [1]. The overproduction of FGF23 culminates in severe renal phosphate wasting, leading to hypophosphatemia. We report a case of undiagnosed TIO in a pregnant woman, along with an assessment of her child’s long-term growth and development.

## Case report

A G2P2002 woman in her 30s began experiencing generalized body pain after her second childbirth, initially in her ribs, which then progressed to both sides of her hips and legs. Over time, her pain intensified from mild to severe. Two years later, the patient was unable to lift her thighs while walking. Despite the escalating pain and lack of specific treatment, she unexpectedly conceived spontaneously. Her pain peaked throughout pregnancy. She rated her pain intensity as 10/10 on the Visual Analog Scale (VAS), even when she received many acetaminophen pills. Even mild breathing made her painful, and the patient felt that she could die at any moment. In addition, she remained mostly sedentary and required assistance walking. Despite these challenging symptoms, her pregnancy persisted without any obstetric complications. After giving birth to a 3200-gram baby boy via cesarean section, the patient struggled with difficulties in daily activities and was unable to hold her newborn for breastfeeding.

When referred to our endocrine clinic, the patient denied any history of trauma or childhood rickets and her family history was unremarkable. Her BMI was 18.6 kg/m^2^ and her vital signs were normal. On examination, she was unable to lift her thighs, and her gait resembled that of a tumbler walking (Video 1). Tenderness over the dorsum of the right foot was noted, without any apparent redness or swelling.

Previous laboratory tests included low total calcium and normal parathyroid hormone (PTH) levels. Old fractures in bilateral ribs, left sacrum, and right foot’s third metatarsal were found on radiography and technetium-99 m bone scan. Furthermore, lumbar spine MRI revealed bone marrow edema in the left posterior superior iliac spine, S2, and S3 vertebrae. The patient had undergone PET/CT scan with 18-FDG from head to pubic bone, showing no signs of tumor. The contrast between the mildly decreased bone density and multiple severe bone fractures, along with her generalized muscle weakness, prompted us to measure her serum phosphate level, which was found to be in a life-threatening range (Table 1). The calculated TmP/GFR ratio was below normal, indicating renal phosphate wasting. A very high FGF23 level confirmed FGF23-mediated hypophosphatemia, suggesting a likely diagnosis of TIO (Table 1). While awaiting the second PET/CT scan, the patient received an oral phospho-soda solution, calcitriol, and meloxicam for pain relief. After four months, to everyone’s surprise, she was pain-free, able to walk and hold her baby.

**Table 1:** Laboratory results of the patient at our endocrine clinic

**Laboratory indexes**	**Result/**	**Normal range**
Ionized Ca^2+^	0.96 mmol/l	1.15–1.33
Serum phosphate	0.26 mmol/l	0.74–1.52
Serum 25(OH)D	27.3 ng/ml	6.6–49.9
Serum creatinine	49.3 μmol/l	50.4–98.1
eGFR	125 ml/min/1.73m^2^	≥90
AST	19 U/l	0–55
ALT	21.45 U/l	5–34
CPK	58 U/l	29–168
Urinary phosphate	14.62 mmol/l	
Urinary creatinine	5.7 mmol/l	
TmP/GFR TRP	0.26 mmol/l 0.69	0.84–1.23
Plasma intact FGF23	484.9 pg/ml	23.2–95.4

**Figure 1:**
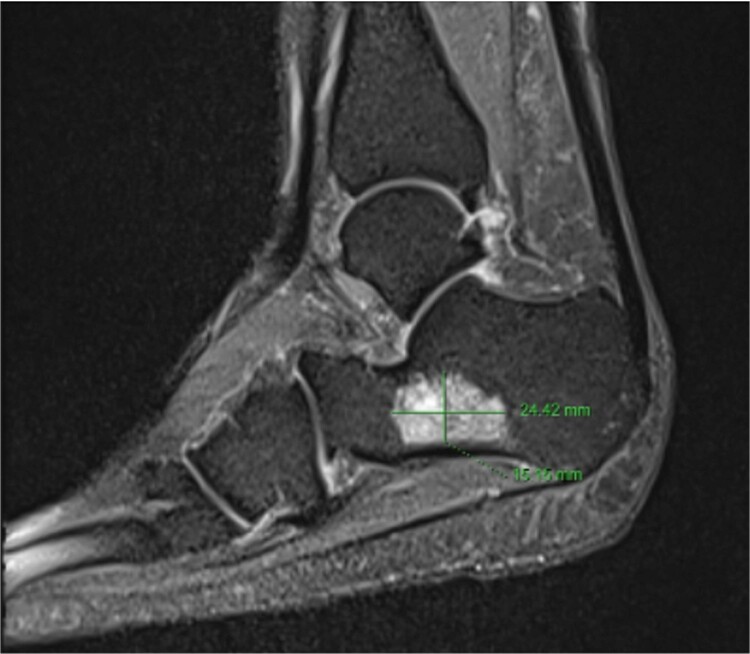
A 15 × 24-mm tumor in the right calcaneus was prominently visible in the MRI images, with irregular margins, strong enhancement, and no invasion beyond the bone.

**Figure 2:**
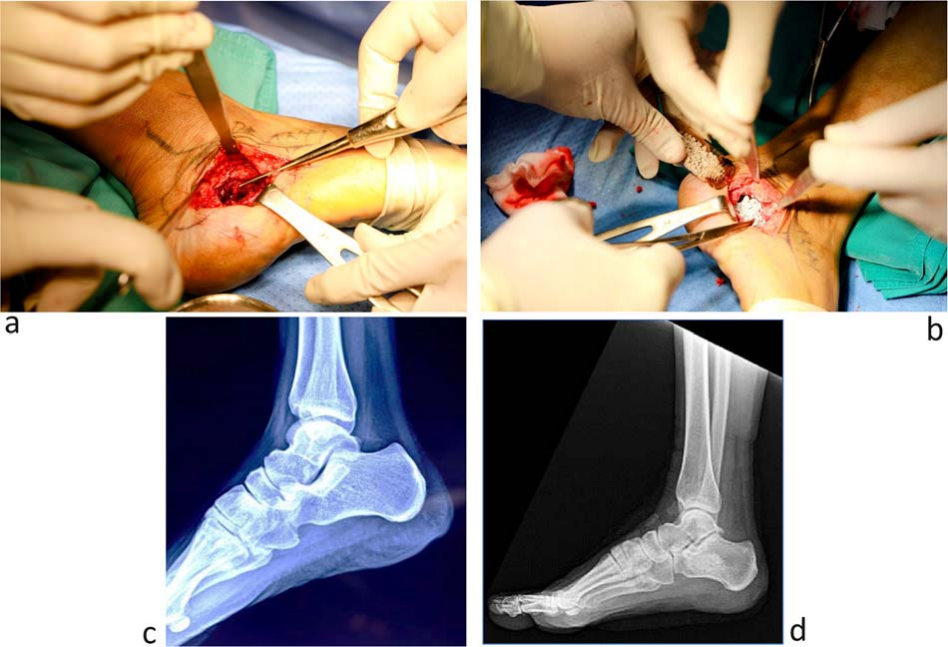
Images taken during surgery (**a** and **b**). A rectangular piece of cortical bone was dissected, revealing an area of tumor with sides 2.5 cm × 1.5 cm × 1.5 cm including yellow gray, poorly demarcated, soft neoplasm surrounded by red spongy bone tissue. A curette was used to remove the tumor and the surrounding irregular tissues until a solid dense cortical bone was reached (**a**). A unit of synthetic bone graft was well-compacted in the socket (**b**). Radiography of the right calcaneus before and one month after surgery (**c** and **d**, respectively) showed that the bone edge was not deformed (**d**).

The second whole-body PET/CT scan using Gallium-68 Dotatate staining revealed an intensely tracer-avid sclerotic lesion in the right calcaneus (SUVmax 33.7, Im 485). Additionally, old healed fractures of the bilateral ribs, right inferior and superior pubic rami were observed. An orthopedist consulted for a foot MRI to plan the surgery (Fig. 1). She underwent wide-margin surgical resection to prevent recurrence (Fig. 2).

**Figure 3:**
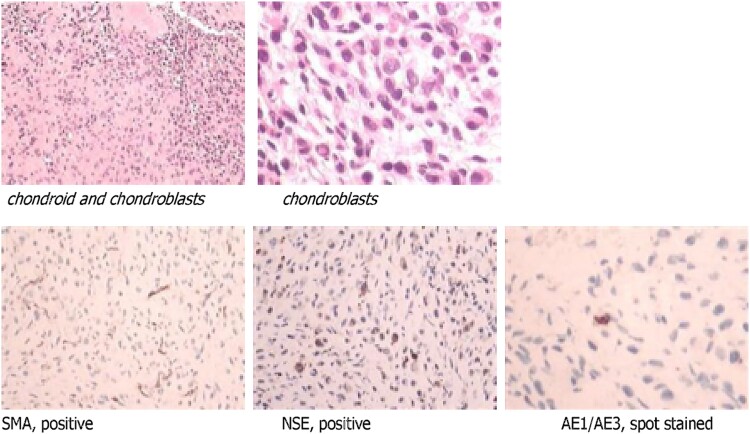
Immunohistochemistry study revealed that the curetted tissue was a chondroblastoma (benign cartilage-forming neoplasm).

At 24 h post-surgery, intact FGF23 level decreased to 24.7 pg/ml, while the tubular reabsorption of phosphate (TRP) rose from 53% preoperatively to 86%. Immunohistochemistry confirmed that the tumor was a chondroblastoma (Fig. 3). One month after surgery, her phosphate and ionized calcium levels remained normal, even though she had stopped taking phospho-soda. However, her 25(OH)D levels remained low, necessitating continued vitamin D3 supplementation (Table 2).

**Table 2:** Laboratory test results of the patient one month postoperatively (at the patient’s local laboratory)

**Laboratory indexes**	**Result/**	**Normal range**
Ionized Ca^2+^	1.2 mmol/l	1.1–1.35
Serum phosphate	1.45 mmol/l	0.81–1.45
Serum 25(OH)D	23 ng/ml	30–40
Serum creatinine	45.78 μmol/l	44–106
eGFR	128 ml/min/1.73m^2^	≥90

**Figure 4:**
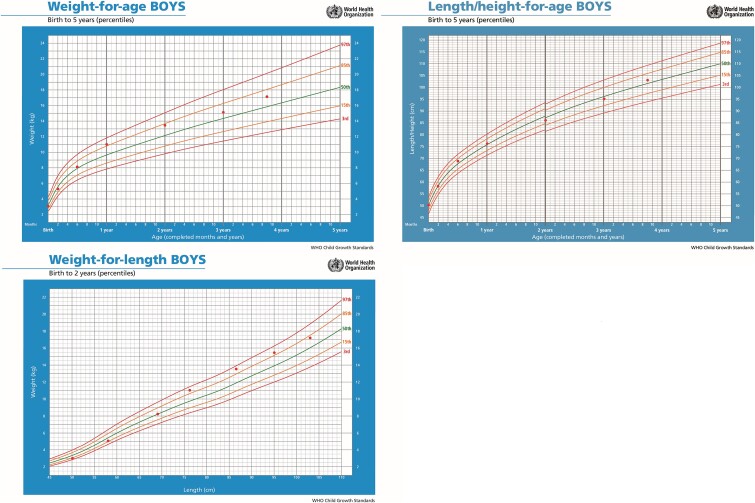
Her child’s growth falls within the normal percentiles for age, according to WHO child growth standards. He grew 26 cm in the first year of life and approximately 10 cm per year over the next three years.

Intriguingly, her child exhibited excellent growth (Fig. 4). The developmental assessment during the first years of life, based on the Denver II developmental screening test, was typical. The child’s gait is normal. He did not complain of any pain or muscle weakness.

## Discussion

Notably, we report a rare case of full-term pregnancy in a woman with undiagnosed and untreated-TIO [2–4]. By the completion of full-term gestation, the typical fetus accumulates approximately 20 g of phosphorus to mineralize its skeleton and support regular physiological functions [5]. The heightened demand for phosphate in the fetus likely worsens maternal hypophosphatemia. At this time, the patient’s blood phosphate has not been measured, but in this context, it was probably very low. This may clarify the reason behind the patient’s excruciating pain during pregnancy. Only three cases of TIO during pregnancy have been reported, one of which involved phosphate and calcitriol supplementation [2–4]. Information regarding these pregnancies is limited. Similarly, while there have been studies on FGF23 in pregnant mice [6, 7] and one report of four babies born to parents with familial hypophosphatemic hyperphosphaturic rickets [8], our case provides the first evidence that TIO during pregnancy does not affect the child’s development and long-term growth in humans.

In any case, leaving a pregnant mother in a severe state of hypophosphatemia is dangerous and requires early detection and treatment. However, managing patients with TIO presents several challenges in clinical practice [9]. The first challenge is that TIO often goes undiagnosed for many years. While doctors are well-versed in bone diseases related to calcium, vitamin D3 metabolism and PTH dysregulation, they may be less familiar with phosphate and FGF23 disorders. The second challenge is that the initial selection of 18-FDG for the PET/CT scan was irrelevant, as the radiologist omitted the lower extremities, despite the lower limbs being the most common site of TIO tumors, as mentioned earlier. This case alerts physicians, including obstetricians and gynecologists, family physicians, and orthopedists, to pay attention to pathologies related to phosphate metabolism, including TIO.

## Consent

Written informed consent was obtained from the patient for publication of this case.

## Guarantor

Luong Dai Ly.

## Supplementary Material

Clip_1_omae125

Video_1_legend_omae125

## References

[1] Bosman A , Palermo A, Vanderhulst J. et al. Tumor-induced Osteomalacia: a systematic clinical review of 895 cases. Calcif Tissue Int 2022;111:367–79. 10.1007/s00223-022-01005-8. 35857061 PMC9474374

[2] Clifton-Bligh RJ , Hofman MS, Duncan E. et al. Improving diagnosis of tumor-induced osteomalacia with Gallium-68 DOTATATE PET/CT. J Clin Endocrinol Metab 2013;98:687–94. 10.1210/jc.2012-3642. 23295468

[3] Sum M , Hoda ST, Rapp T. et al. Tumor-induced Osteomalacia localized and excised after pregnancy. AACE Clin Case Rep 2021;7:363–6. 10.1016/j.aace.2021.06.008. 34765732 PMC8573288

[4] Muniz CR , Bezerra GAM, da Silva VC. et al. Ethmoid glomangioma and oncogenic osteomalacia: a case report. J Med Case Rep 2021;15:348. 10.1186/s13256-021-02916-0. 34271987 PMC8285823

[5] Kovacs CS . Calcium and Phosphate Metabolism and Related Disorders During Pregnancy and Lactation. In: Endotext. Feingold KR, Anawalt B, Blackman MR. et al. eds. MDText.com, Inc Copyright © 2000-2024: South Dartmouth (MA), 2000. 25905396

[6] Ma Y , Samaraweera M, Cooke-Hubley S. et al. Neither absence nor excess of FGF23 disturbs murine fetal-placental phosphorus homeostasis or prenatal skeletal development and mineralization. Endocrinology 2014;155:1596–605. 10.1210/en.2013-2061. 24601885 PMC3990847

[7] Ohata Y , Yamazaki M, Kawai M. et al. Elevated fibroblast growth factor 23 exerts its effects on placenta and regulates vitamin D metabolism in pregnancy of Hyp mice. J Bone Miner Res Off J Am Soc Bone Miner Res 2014;29:1627–38. 10.1002/jbmr.2186. 24470103

[8] Moncrieff MW . Early biochemical findings in familial hypophosphataemic, hyperphosphaturic rickets and response to treatment. Arch Dis Child 1982;57:70–2. 7065698 PMC2863253

[9] Brandi ML , Clunie GPR, Houillier P. et al. Challenges in the management of tumor-induced osteomalacia (TIO). Bone 2021;152:116064. 10.1016/j.bone.2021.116064. 34147708

