# Supplementary material for: Full-term pregnancy despite severe hypophosphatemia caused by tumor-induced osteomalacia
Source: Oxf Med Case Reports. 2024 Oct 26;2024(10):omae125. doi: 10.1093/omcr/omae125 (PMC11512694; doi:10.1093/omcr/omae125)
Supplement: Video_1_legend_omae125 [file video_1_legend_omae125.docx]

**Video 1.** The patient was unable to lift her thighs while walking and moved unsteadily, which resembled a tumbling motion.
